# A Comparative Study of Quercetin-Loaded Nanocochleates and Liposomes: Formulation, Characterization, Assessment of Degradation and In Vitro Anticancer Potential

**DOI:** 10.3390/pharmaceutics14081601

**Published:** 2022-07-31

**Authors:** Neha Munot, Ujjwala Kandekar, Prabhanjan S. Giram, Kavita Khot, Abhinandan Patil, Simona Cavalu

**Affiliations:** 1 Department of Pharmaceutics, School of Pharmacy, Vishwakarma University, Pune 411048, Maharashtra, India; 2 Department of Pharmaceutics, JSPMs Rajarshi Shahu College of Pharmacy and Research, Tathwade, Pune 411033, Maharashtra, India; ujja2303@gmail.com; 3 Department of Pharmaceutics, Dr. D.Y. Patil Institute of Pharmaceutical Sciences and Research, Pimpri, Pune 411018, Maharashtra, India; prabhanjanpharma@gmail.com; 4 Department of Pharmaceutical Sciences, University at Buffalo, The State University of New York, Buffalo, NY 14214, USA; 5 Department of Pharmaceutics, Sinhgad Technical Education Society’s Smt. Kashibai Navale College of Pharmacy, Pune 411048, Maharashtra, India; kavikhot08@gmail.com; 6 Department of Pharmaceutics, School of Pharmacy, Sanjay Ghodawat University, Kolhapur 416118, Maharashtra, India; abhinandan.patil@sanjayghodawatuniversity.ac.in; 7 Faculty of Medicine and Pharmacy, University of Medicine, P-ta 1 Decembrie 10, 410087 Oradea, Romania

**Keywords:** nanocochleates, liposomes, quercetin, dimyristoyl phosphatidyl glycerol, human mouth cancerKB cell lines, rat liver homogenate (S9G)

## Abstract

Quercetin, a flavonoid, has antioxidant and anti-inflammatory properties and the potential to inhibit the proliferation of cancer, but its therapeutic efficacy is lowered due to poor solubility and bioavailability. Quercetin-loaded nanocochleates (QN) were developed using a trapping method by the addition of calcium ions into preformed negatively charged liposomes (QL) prepared by a thin-film hydration method. Liposomes were optimized by varying the concentration of Dimyristoyl phosphatidyl glycerol and quercetin by applying D-optimal factorial design using Design-Expert^®^ software. Stable rods were observed using TEM with an average particle size, zeta potential and encapsulation efficiency of 502 nm, −18.52 mV and 88.62%, respectively, for QN which were developed from spherical QL showing 111.06 nm, −40.33 mV and 74.2%, respectively. In vitro release of quercetin from QN and QL was extended to 24 h. Poor bioavailability of quercetin is due to its degradation in the liver, so to mimic in vivo conditions, the degradation of quercetin released from QL and QN was studied in the presence of rat liver homogenate (S9G) and results revealed that QN, due to its unique structure, i.e., series of rolled up solid layers, shielded quercetin from the external environment and protected it. The safety and biocompatibility of QL and QN were provenby performing cytotoxicity studies on fibroblast L929 cell lines. QN showed superior anticancer activity compared to QL, as seen for human mouth cancerKB cell lines. Stability studies proved that nanocochleates were more stable than liposomal formulations. Thus, nanocochleates might serve as pharmaceutical nanocarriers for the improved efficacy of drugs with low aqueous solubility, poor bioavailability, poor targeting ability and stability.

## 1. Introduction

Cancer is emerging as a major health issue globally and also exhibits many challenges in its treatment. It is associated with a high mortality rate and crucial hurdle to life expectancy, irrespective of developing and developed countries. Statistical data predict that more than 100 types of cancers were reported so far, inclusive of breast, rectal, prostate, liver and lung cancer. An estimated 19.3 million new cases of cancer and nearly 10 million cancer deaths occurred worldwide in 2020 [1]. Regardless of new therapeutic approaches being progressively developed, effective cancer treatment therapies are still in demand [2]. Recently, it has been noted that a diet enriched with fruits and vegetables can lower the risk of cancer [3,4]. Quercetin is commonly found in vegetables and fruits in the form of a glycoside with an exceptionally high concentration in onions, apples, tea, broccoli and red wine [5]. The chemical name of quercetin is 3, 3′, 4′, 5, 7-pentahydroxyflavone. Quercetin suppresses numerous tumor-related activities, mainly apoptosis, oxidative stress, proliferation and metastasis, hence it was categorized as a potential chemo preventer [6]. It has several mechanisms, such asthe restriction of mutant p53 expression and augmentation of death-receptor-mediated apoptosis in glioma cells [7], inhibits cancer as a consequence of oxidative stress [8,9,10], metastasis and apoptosis against tumor cell lines [11,12,13], inhibits the activity and expression of P-glycoprotein in ADR/MCF-7 (Adriamycin resistant/Michigan Cancer Foundation-7) cells, remarkable resistance of doxorubicin in ADR/MCF breast cancer cells that promote immune responses against breast tumor propagation [14], quercetin in combination with SN-38 (an active metabolite of irinotecan) shows viability of AGS (A human gastric adenocarcinoma cell) cells and the proportion of apoptosis comparable to treatment of high-dose SN-38 alone [15], anti-proliferative activity of quercetin which is mediated by type II estrogen binding site, which is resistant to tamoxifen [16], induces pro-apoptotic autophagy via SIRT1/AMPK signaling pathway, etc. [17].

The interesting therapeutic potential of quercetin is unfortunately limited to its limited oral bioavailability of about 4%, due to its high lipophilicity and half-life of 3.5 h. Poor bioavailability and therapeutic response due to the rapid metabolism of the drug necessitates high dose and frequent administration leading to patient incompliance [18,19]. To overcome these limitations, various formulations have been developed for quercetin such as microemulsions, self-micro and nano-emulsifying drug delivery systems, phytosomes, micelles, solid lipid nanoparticles, liposomes, etc. [20,21,22,23,24,25]. Despite these innovations, most of these formulations suffer from less mechanical stability, restricted drug-loading abilities, liposomes are not stable after lyophilization and may cause the leakage of the drug upon storage for a long time. Thus, there is a need to address these problems and develop formulation that would overcome the abovementioned limitations of other nanocarriers.

Nanocochleates are precipitates of stable phospholipids that are the result of binding between anionic lipid vesicles and divalent cations such as Ca^2+^ and Mg^2+^. They do not have internal aqueous spaces and are composed of a uniform, solid-lipid bilayer sheath rolled in a supraspiral fashion [26]. Nanocochleates have a number of benefits, such as the ease and safety of scale up, they are accepted by the body, they have reduced side effects and higher efficacy. Thus, they have the potential to be used in the development of novel pharmaceuticals [27]. These novel carriers have been successfully investigated for the delivery of several classes of drugs including antibiotics, antifungal, anti-leprosy, anticancer, protein and DNA subunit, etc. to improve their therapeutic efficacy [28,29,30,31]. The potential for nanocochleatesto be used as carriers for anticancer drugs has been discussed by Nayeket al. as these novel lipid-based carriersare more stable due to less oxidation of lipids. These are non-immunogenic, non-inflammatoryandnon-toxic. They have the ability to interact with cancerous cells, so that they can deliver anticancer drugs effectively [32]. Sonawane et al. performed preliminary studies on nanocochleates loaded with flavonoids, such as quercetin, to analyze whether this nanocarrier can be used in drug delivery in clinical settings [33].

In the present investigation, systematic approaches and elaborate studies have been carried out to develop quercetin nanocochleates. These were developed through conversion of Dimyristoyl phosphatidyl glycerol (DMPG) and cholesterol multilamellar liposomal vesicles into the cochleate. The studies further evaluated and compared quercetin-loaded liposomes and pure quercetin solution.

## 2. Materials and Methods

### 2.1. Materials

Quercetin was purchased from Sigma-Aldrich (Mumbai, India). Cholesterol was procured from Research-Lab Fine Chem Industries Ltd. DMPG was procured from Lipoid GmbH Ludwigshafen, Germany. Ethylene diaminotetraacetic acid (EDTA) was procured from the Research Lab. Calcium chloride was procured from Ana Lab Fine chemicals. Polyethylene glycol 400 (PEG 400), chloroform, sodium phosphate (dibasic), potassium phosphate (monobasic), sodium hydroxide, methanol and acetonitrile (HPLC grade) were purchased from Loba Chemicals. Dimethylsulfoxide (DMSO) was procured from Merck Chemicals, Mumbai, India. Dialysis bag 80 (molecular weight cut off 12,000) was purchased from Sigma-Aldrich Chemical Private Ltd. (Bangalore, India). Rat liver homogenate- S9G was generously gifted by Toxindia, Pune, India.

### 2.2. Preparation of Quercetin-Loaded Liposomes

Step 1

The thin-film hydration method with a slight modification was used to prepare quercetin-loaded liposomes [34]. Briefly, different ratios of DMPG and cholesterol were dissolved in the chloroform:methanol solvent mixture (2:1 *v*/*v*) and then 5 mg of quercetin was added into this mixture which was introduced into the 250 mL round bottom flask with a ground-glass neck. This flask was then attached to the rotary evaporator (Superfit 90 Rotavap Model: PBCT-8D, India) and rotated at 60 rpm. The organic solvents were evaporated at about 40 °C. The pressure at the cylinder head was gradually raised until there was no difference between the inside and outside pressure of the flask. A thin film was formed on the inside surface of the flask after 15–30 min.

Step 2

The flask was then flushed with 10 mL distilled water and attached again to the rotary evaporator and rotated at room temperature and pressure at the speed of 60 rpm. The flask was left to rotate for 60 min, after which a homogenous suspension was formed. This suspension was allowed to standovernight at 4 °C. Due to the hydration of lipids, spherical vesicles were formed [34,35]. Different batches were prepared by varying the concentrations of DMPG and quercetin by applying D-optimal factorial design (Table 1). The design was obtained by Design-Expert^®^ (Version 11, Stat-Ease Inc., Minneapolis, MN, USA). The effect of DMPG and drug concentration on the size of the particles and entrapment efficiency was evaluated.

### 2.3. Preparation of Quercetin-Loaded Nanocochleates

Nanocochleates were prepared from preformed liposomes using a trapping method. Optimized batches of liposomes were selected for the formulation of nanocochleates. For the formation of cochleates, 50 µL calcium chloride solution (0.1 M) was added dropwise to quercetin-loaded liposomal vesicles under vortex using a probe sonicator (Spectra lab model UCB 40, India). The vesicle phase immediately turned turbid, indicating the formation of nanocochleates. Precipitated nanocochleates were refrigerated at 2–8 °C (Table 2) [36].

### 2.4. Particle Size Analysis

The size of the particles of quercetin-loaded liposomes (QL) and quercetin-loaded nanocochleates (QN) was studied by a particle size analyzer (Sympatec-Nanophox-NX0088, Germany). This technique is based on the principle of photon cross-correlation spectroscopy. The sample was diluted with distilled water, filled in the transparent cuvette and placed in a thermostat water bath, which was maintained at 25 °C. The laser beam at a scattering angle of 90° was incident on the sample and particles in the sample underwent Brownian motion. The speed of a measured particle converted into a hydrodynamic diameter using the Stokes–Einstein equation [37]. Average particle size was measured in triplicate.

### 2.5. Zeta Potential Measurement

Charge on the surface of QL and QN was determined using DelsaNanoC zeta potential analyzer (Beckman Coulter, Brea, CA, USA). Analysis time was kept to 1 min and the average zeta potential and charge of QN and QL were determined. The temperature was set to 25.2 °C and 3 runs were carried out.

### 2.6. Determination of Encapsulation Efficiency (EE) of QL

Entrapment efficiency was calculated by isolating non-encapsulated quercetin from vesicular suspension of liposomes by centrifugation (Kubota lab centrifuge Japan) at 12,000 rpm for 2 h at 4 °C. The sediment vesicles were disordered with ethanol to release the entrapped drug. These were appropriately diluted with ethanol, absorbance was noted at 373 nm using UV Spectrophotometer (Jasco V-630) [38]. Initially, a calibration curve (R^2^ = 0.999) was obtained by measuring the absorbance of the quercetin solutions (concentration of 1 to 20 μg/mL in ethanol) at 373 nm. The equation forthe calibration curve was: y = 0.0739x + 0.0491. ‘y’ and ‘x’ werethe absorbance measured and concentration in μg/mL, respectively. The EE (%) was derived using Equation (1).

EntrapmentEfficiency (%) = (Amount of drug entrapped in QL)/(Total amount of drug present) × 100(1)

### 2.7. Determination of Encapsulation Efficiency of QN

QN (100 µL) was added to a centrifuge tube made up of polypropylene, which was centrifuged at 6000 rpm for 20 min at 40 °C, and the supernatant and sediment separated. In total, 60 μL of EDTA (pH 9.5) was added to the QN sediment to allow the escape of quercetin from the cochleates. Ethanol (1 mL) was added to the above mixture for further extraction of the drug. The resulting clear solution was appropriately diluted with phosphate buffer pH 7.4 and absorbance was determined at 373 nm using a UV spectrophotometer (Jasco V-630). Concentration of the free drug in the supernatant was measured and EE was obtained using Equation (2) [39]

EE (%) = (Amount of drug entrapped in QN)/(Total amount of drug present) × 100(2)

### 2.8. Surface Morphology

The surface morphology of QL and QN dispersion was studied by transmission electron microscopy (TEM). TEM sample was prepared by mixing 20 µL of formulation and 20 µL of 1% phospho tungstic acid in an Eppendorf tube. A drop of the diluted sample was positioned on a carbon-coated copper grid to generate the thin film of liquid. The sample was observed and photographed with a transmission electron microscope (TECNAI E, Germany) [40].

### 2.9. Differential Scanning Calorimetry (DSC)

Differential scanning calorimeter (Perkin Elmer, Waltham, MA, USA) was employed to perform DSC measurements. An appropriately weighed quantity (10 mg) was added in a sealed aluminum pan, under the atmosphere of nitrogen flow (20 mL/min) at a scanning rate of 20 °C per min in the range of 100–300 °C. An empty aluminum pan was used as a reference [40].

### 2.10. In Vitro Release of Quercetin from QL and QN

In vitro release studies of quercetin from QL and QN wereperformed in phosphate-buffered saline (pH 7.4) by adopting a dialysis bag diffusion method and the results were compared with a pure solution of quercetin (1 mg/mL in 30% *w/w* polyethylene glycol 400 and water). Formulations QL7 and QN3 equivalent to 1 mg of quercetin were placed into a dialysis bag (cellulose membrane, molecular weight cut off 120,000 Da), sealed thoroughly and submerged into 100 mL of dissolution medium. The assembly was maintained at 37 ± 0.5 °C with constant stirring by a magnetic stirrer at 100 rpm. At predetermined intervals, the samples were withdrawn and an equal volume of fresh medium was added to achieve sink conditions. The absorbance of quercetin in the solution was determined using the double beam UV-Vis spectrophotometer (Jasco V-630, Japan) [41].

### 2.11. Degradation Studies of Quercetin in the Presence of Rat Liver Homogenate (S9G)

To mimic in vivo conditions and to study the degradation of quercetin released from QL7 and QN3, rat liver homogenate (S9G) was added to the release medium i.e., phosphate-buffered saline (PBS, pH 7.4) and the results were compared with pure quercetin solution. This study was performed using the dialysis bag diffusion method with slight modification. Formulation equivalent to 1 mg of quercetin and 1 mg quercetin solution (1 mg/mL in 30% *w/w* mixture of polyethylene glycol 400 and water) as a control was added to a dialysis bag (cellulose membrane, molecular weight cut off 120,000 Da), sealed and submerged into 100 mL of dissolution medium containing rat liver homogenate-S9G (Obtained as a gift sample from Intox India Ltd., Pune, India). The assembly was maintained at 37 ± 0.5 °C with constant stirring by a magnetic stirrer at 100 rpm. At predetermined intervals of 1 h and 8 h, aliquots (5 mL) were withdrawn and an equal volume of fresh dissolution medium was added to maintain sink conditions. These aliquots (20 µL) were injected into the HPLC System (JASCO LC–NET II/ADC, Japan).

The HPLC settings for the analysis of quercetin were: pump: PU-2080 (JASCO, Japan); injector: an autosampler (AS-1555); column: HiQSil C18HS, 250 × 4.6 mm, 5 µ with a Javelin Guard column (10 × 4.6 mm. 5 µ); and detector: PDA (photodiode array detector (JASCO). Methanol at pH 5 adjusted with ortho-phosphoric acid (99% *v/v*) was selected as a mobile phase. The temperature of the column was 27 °C and a flow rate of 1 mL/min was maintained with a detection wavelength of 373 nm. The retention time of quercetin was found to be at 2.6 min at these conditions. The LOD and LOQ valueswerefound to be 0.1206 and 0.365 ppm, respectively. The calibration curve was linear (y = 592.2x + 326.6) with its correlation coefficient being 0.999.

### 2.12. Cytotoxicity Study

Cytotoxicity studies were carried out usingthe method reported by Cobanet al. (2019) with certain modifications on healthy L929 fibroblast cell lines, performed using a cell viability assay. The free drug, QN1 and QL2 were sterilized by 30 min UV exposure inside the biosafety cabinet. The free drug was initially dissolved in sterile DMSO and further dilutions were carried out using complete Dulbecco’s modified Eagle medium (DMEM). QL1 and QN2 were diluted using complete DMEM. Healthy L929 fibroblast cell lines (passage number 59) were conserved using complete DMEM. Fibroblast cells (10,000) were plated in each well of a 96-well plate. It was incubated in 5% CO_2_ at 37 °C for 1 day. After 24 h, samples of 1.53, 3.0265, 6.125, 12.5, 25 and 50 µM concentrations were added in triplicates. Later resazurin solution (in complete DMEM media) was incorporated into the well. It was incubated for 6 h in 5% CO_2_ at 37 °C. In total, 100 μLof media from each well was taken and read in a plate reader (excitation 530 to 560 nm and emission at 590 nm) [40].

### 2.13. In Vitro Anticancer Activity

In total, 5000 KB cells (human mouth cancer, passage number 402/403) were seeded in each well of a 96-well plate. Pure quercetin, QN and QL were dissolved in DMSO. Different concentrations of these samples containing 2.5, 5, 10, 20, 30 and 40 μg/mL of drug in 100 μL complete media were added and they were incubated for 1 and 2 days under cell culture condition (10% Minimum essential medium (MEM), 5% CO_2_ at 37 °C). Ten microliters of MTT (5 mg/mL) reagent in MEM media wasaddedintoeach well and re-incubated for another 3 h at 37 °C. Formazan crystals were solubilized in 100 μL DMSO and added to each well. After 10 min, the absorbance was recorded at 570 nm. Percent mitochondrial activity was calculated by Equation (3).

Percent mitochondrial activity = (Absorbance at 570 nm of treated samples/Absorbance at 570 of untreated samples) × 100(3)

### 2.14. Stability Studies

Freshly prepared quercetin-loaded liposomal and nanocochleate suspensions westored at 5 °C ± 3 °C for three months and the effect on various parameters such as particle size and morphology, zeta potential and entrapment efficiency was studied. Additionally, lyophilized formulations of liposome QL7 and nanocochleates QN3 were placed into amber-colored glass vials, sealed and stored according to the abovementioned conditions, as per the ICH QA1 R2 guidelines [42].

## 3. Result and Discussion

Quercetin-loaded nanocochleates (QN) were formulated using the trapping technique through the addition of calcium ions into preformed negatively charged liposomes (QL) comprising dimyristoyl phosphatidyl glycerol (DMPG) and cholesterol. D-optimal design was used to analyze the effect of DMPG and quercetin on entrapment efficiency and particle size. Design-Expert^®^ (Version 11, Stat-Ease Inc., Minneapolis, MN, USA) was used to generate the design and carry out the evaluation by statistical means. Main variables, i.e., DMPG and the amount of quercetin, had equal impact on particle size and entrapment efficiency; thus, the effect of these parameters was evaluated by the analysis of variance (ANOVA) partial sum of squares or Type III. This type of ANOVA is used to consider the equal effect of main variables at selected levels. The same test was also suggested by the Design-Expert^®^ software. In connection with these variables, the 2FI (two factor interaction) model was selected to analyze the response. The 2FI model denoted the interaction of the main variables, mainly DMPG and quercetinat selected levels to alter the entrapment efficiency and particle size. Hence, the major effect of these variables was studied using 2FI model.

DMPG is one of the essential components of the mammalian cell membrane and was found to possess better compatibility for human use [43]. Moreover, negatively charged DMPG was chosen as the phospholipid component that had the potential to react with positively charged calcium ions to form stable cochleates. Cholesterol was included to form complexes with drugs as well as to stabilize the phospholipid membrane [26]. Calcium ions were chosen for the formulation of nanocochleates. Calcium ions were held in the center and connected with the anionic lipid head of one bilayer as well as the opposite bilayer, resulting in a planar sheet, which ultimately coiled near theinitial point of folding to generate rod-shaped structures which are called cochleates [41]. Due to the unique structure of nanocochleates, it allows maximum encapsulation of the free drug [29]. Ca^2+^ was selected as a positively charged ion instead of Ba^2+^, Mg^2+^ and Zn^2+^, as it could alter the phases of the bilayer membrane to initiate membrane fusion naturally; in addition, it forms a less hydrated tightly packed structure at a lower concentration than Mg^2+^ [44,45]. Moreover, it is universal fact that Ca^2+^ has a crucial function in natural membrane fusion, whereas Ba^2+^, Mg^2+^ and Zn^2+^ were not as useful as Ca^2+^. Ultimately, it is better suited to human physiology [46].

### 3.1. Formulation and Evaluation of Quercetin-Loaded Liposomes

Thin-film hydration method was used for the preparation of liposomes as this method had been widely used due to the ease of handling [47]. The different batches were prepared by varying the concentration of DMPG and cholesterol as shown in Table 1. For the preparation of liposomes, the amount of quercetin and phospholipid was varied as 5, 10, 15 mg and 30, 50 and 70 mg, respectively. The size of the vesicles increased asthe amount of the drug increased; on the other hand, as thedrug concentration increased, the EE reduced. These results might be attributed to an insufficient amount of phospholipid to entrap the drug. Hence, we decided to use the lowest amount of the drug (5 mg) for the further preparation of nanocochleates. These results were in agreement with the research carried out by Bothira et al. (2018), where the researcher had chosen 3, 5 and 7 mg of doxycycline in the liposomal formulation and optimized 5 mg of the doxycycline for incorporation into nanocochleates [26]. In the present study, the formulations of QL1, QL4 and QL7 containing 5 mg of quercetin had better entrapment efficiency, zeta potential, particle size and sphericity of particles (Table 4). Therefore, these were considered optimized batches of liposomes and were converted to nanocochleates.

### 3.2. Preparation of Quercetin-Loaded Nanocochleates

The trapping method was used for the preparation of nanocochleates. Optimized batches of liposomes were selected for the formulation of nanocochleates as shown in Table 2. Liposomes with a 5 mg loading capacity were converted into nanocochleates by the addition of CaCl_2_ solution (0.1 M). The effect of change in the volume of calcium chloride solution (10 µL, 50 µL and 100 µL) was studied. The concentration of CaCl_2_ was much lower than the studies carried out by Liu et al. (2017) [48]. CaCl_2_ (10 µL, 0.1 M) was not sufficient to convert all liposomes into nanocochleates. No difference was observed when 50 µL and 100 µL of CaCl_2_ were added into prepared liposomes; thus, 50 µL CaCl_2_ (0.1 M) was fixed for the formation of nanocochleates. D-optimal design was selected to evaluate the effect of DMPG and quercetin on entrapment efficiency and particle size. This design contains categorical factors that produce a design that is much closer to the full factorial design and can handle the generic design [49]. The response obtained is shown in Table 3.

The 2FI model was appropriate for both the response and model terms, which were found to be significant. Mathematical equations for % EE and size of the particle can be calculated using Equations (4) and (5).

(4) $$\begin{array}{ll} Y1= & 61.61-8.28\times A[1]+4.22\times A[2]+10.56\times B[1]+0.39\times B[2]+6.11\times A[1]B[1]\\ & -3.89\times A[2]B[1]+1.28\times A[1]B[2]-0.22\times A[2]B[2] \end{array}$$

(5) $$\begin{array}{ll} Y2= & 210.11+53.56\times A[1]-67.78\times A[2]-52.11\times B[1]+28.22\times B[2]+1.44\times A[1]B[1]\\ & +59.78\times A[2]B[1]+53.11\times A[1]B[2]-38.56\times A[2]B[2] \end{array}$$

**Figure 1:**
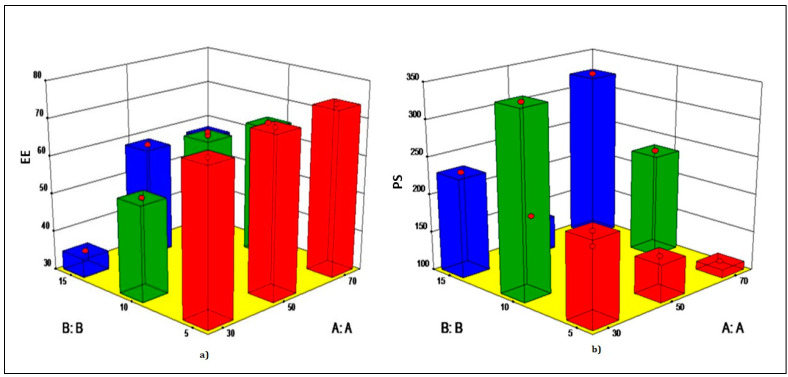
Three-dimensionalsurface graph for liposomes. (**a**) Entrapment efficiency; (**b**) Particle size. A:A—concentration of DMPG. B:B—concentration of quercetin.

Y1 is the entrapment efficiency; Y2 is the particle size; and A and B are the concentrations of DMPG and quercetin, respectively. The above equation depicts that the lower concentration of DMPG had a negative effect on % EE and a positive effect on particle size. Higher concentration of DMPG had a positive impact on both the responses. Higher concentration of the quercetin in combination with a higher amount of DMPG had a negative effect on both the parameters. Higher amount of DMPG and a lower amount of quercetin had a positive impact on both the parameters. Three-dimensional surface graph for entrapment efficiency and particle size (Figure 1) denoted that the minimum level of quercetin (5 mg) and the maximum level of DMPG (70 mg), i.e., formulation Q7, were suitable for nanocochleate preparation. As observed from Table 4 and Table 5, the entrapment efficiency increased as the DMPG concentration increased. The maximum entrapment of drug was seen in the nanocochleates of batch QN3, as the amount of lipid was sufficient to entrap 5 mg drug.

### 3.3. Particle Size and Entrapment Efficiency Determination of Liposomes and Nanocochleates

**Figure 2:**
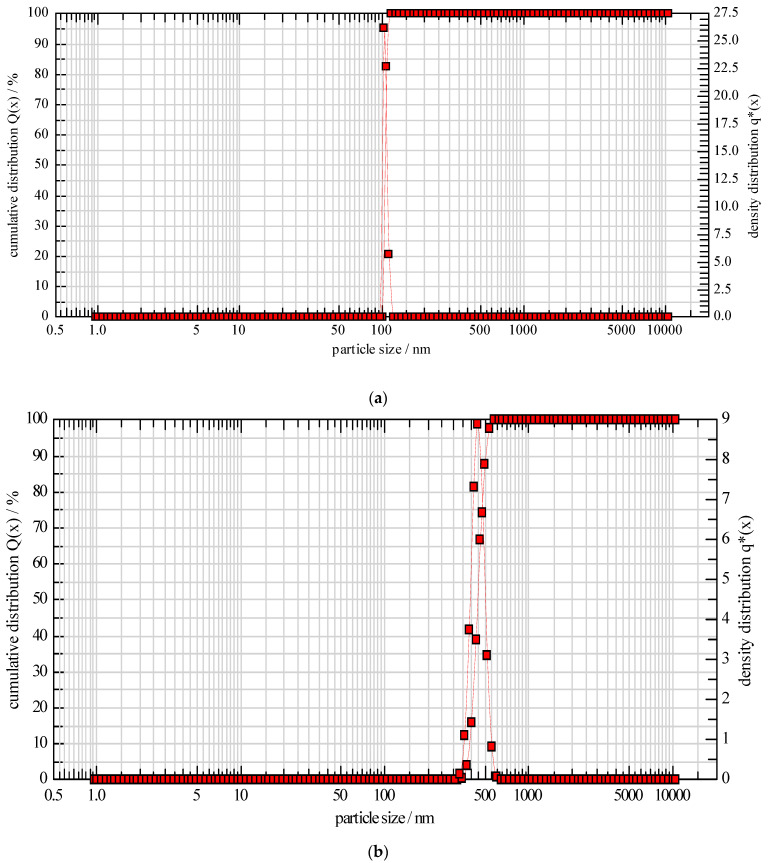
Particle Size of Quercetin loaded (**a**) Liposomes (QL7) and (**b**) Nanocochleates (QN3).

Particle size and polydispersity index were of crucial importance during the formulation of lipid-based nanocarriers as these majorly affect the appearance, process ability, performance and stability of the final product [50]. The mean particle size of formulation QL7 and QN3 was found to be 111.06 ± 2 nm and 502 ± 4 nm (Figure 2). It was observed that liposomes were smaller in size than nanocochleates, as nanocochleates were rod-shaped. These results were inconsistent with the studies carried out by Cobanet et al. (2019) [40]. Polydispersibility index (PdI) ranging from 0 to 1 reflects the polydispersity index of the suspension with the lower value in the range of 0.05 to 0.7, indicating a highly monodispersed suspension. For lipid-based carriers such as liposomes and niosomes, a value of Pdl below 0.3 is acceptable for homogenous phospholipid vesicles [51]. PdI of QN3 and QL7 was found to be 0.09 and 0.33, respectively, indicating the formation of monodispersed suspension. Encapsulation efficiency was important for enhancing the bioavailability of the drug. It was found that the entrapment efficiency increased with an increased amount of polymer and reduced with the increased drug concentration. These results might be attributed to the complete encapsulation of the drug within the bilayers at lower concentrations. An enhanced drug amount resulted in an insufficient amount of lipid to entrap the drug. These results were in agreement with the studies carried out for the entrapment of α-tocopherol in liposomes, where results suggested that up to 5% incorporation of α-tocopherol in vesicles showed enhanced encapsulation efficiency and an increase in concentration reduced the entrapment efficiency [52]. The large size of the nanocochleates meant that they could incorporate a largerdrug amount as seen from the increased entrapment efficiency of QN3 compared to QL7 (Table 4 and Table 5).

### 3.4. Zeta Potential Measurements

**Figure 3:**
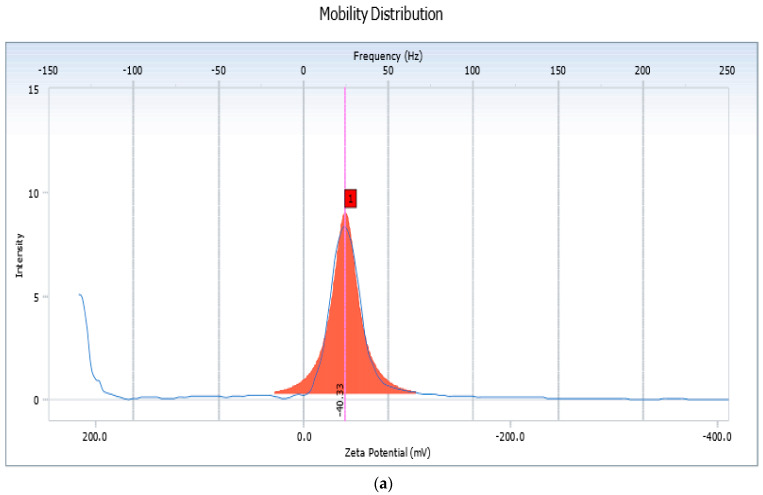

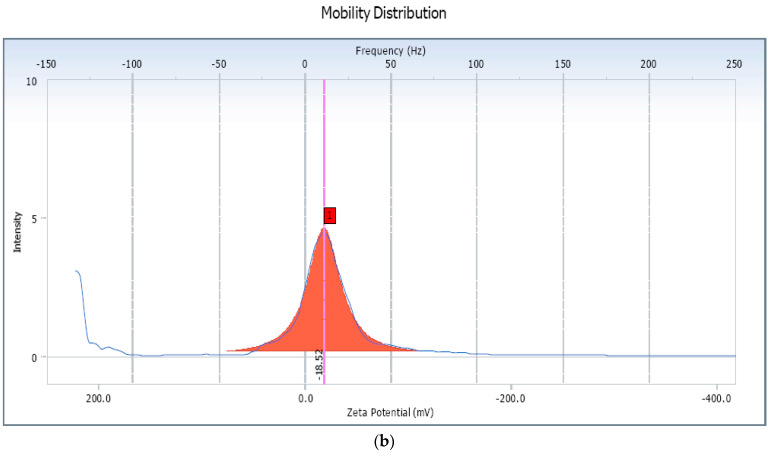
Zeta Potential of Quercetin loaded (**a**) Liposomes (QL7) and (**b**) Nanocochleates (QN3).

Zeta potential measurement is a marker of stability of a colloidal system. The measurement indicates the overall surface charge of a particle and provides information as to whether the system may remain stable or consequently undergo aggregation or flocculation [53]. As depicted in Figure 3, the zeta potential of the liposomes (QL7) and that of the nanocochleates (QN3) was found to be −40.33 mV and −18.52 mV, respectively. These results indicated that prepared liposomes and nanocochleates exhibited sufficient charge to avoid the aggregation of the vesicle. The negative zeta potential value was probably due to the anionic nature of the lipid (DMPG). The incorporation of Ca^2+^ to liposomes encouragesthe fusion of the lipidmembrane, resulting in thegeneration of planar sheets, which ultimately coil near theinitial point of folding to generate rod-shaped cochleates and thus the alteration of zeta potential was observed [54]. Thus, based on the results of particle size, zeta potential and entrapment efficiency, (Table 5), formulation QN3 was considered an optimized batch and was evaluated further.

### 3.5. Surface Morphology

**Figure 4:**
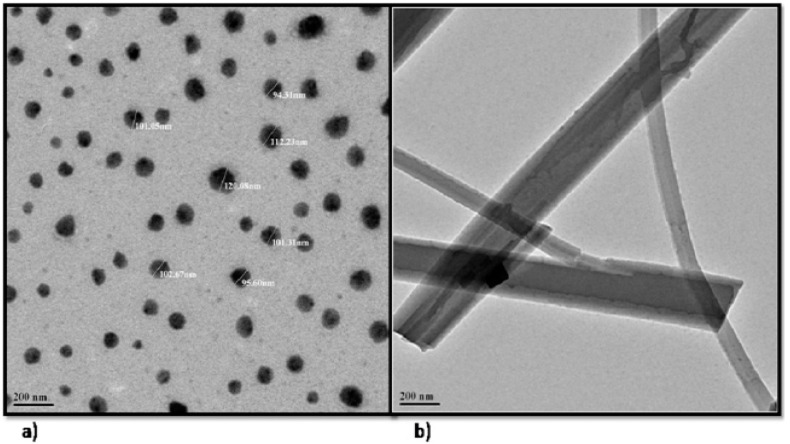
TEM images of (**a**) liposomes and (**b**) nanocochleates.

**Figure 5:**
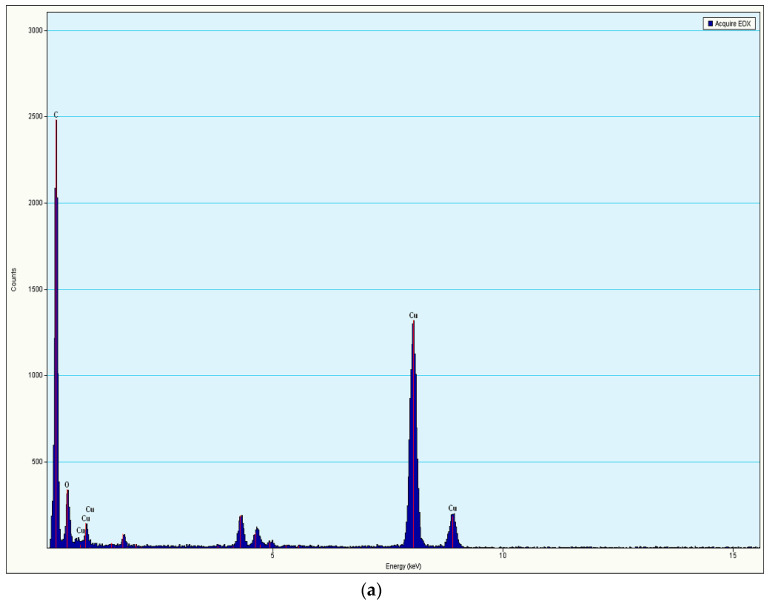

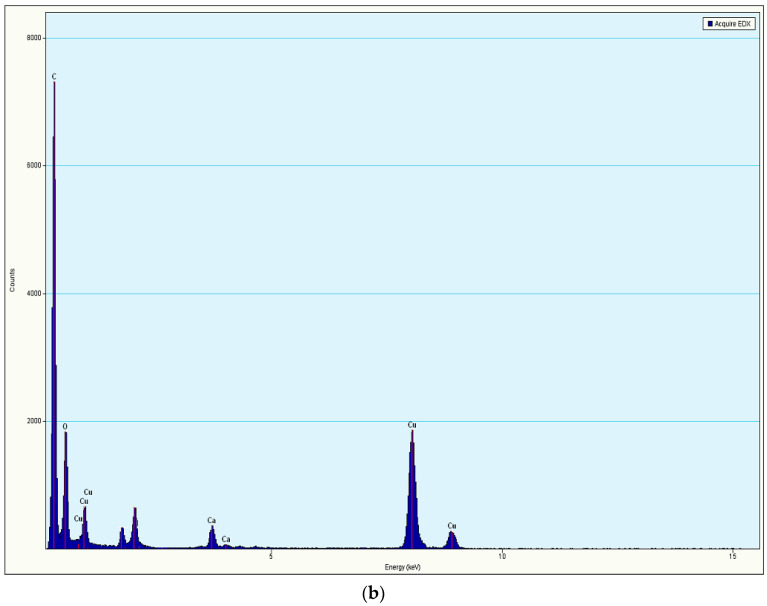
Energy-dispersive X-ray spectra (counts vs. energy) of (**a**) liposomes and (**b**) nanocochleates.

TEM analysis was used to determine the structural and internal properties as these were equipped with high spatial and atomic resolution [55]. The TEM (Figure 4b) confirmed the presence of rolled-up elongated tubular structures of cochleates developed from spherical vesicles of liposomes (Figure 4a). Energy-dispersive X-ray (EDAX) spectra of QL and QN showed characteristic peaks of carbon and oxygen elements indicating the purity of the formulations (Figure 4). The intensity of carbon and oxygen in EDAX spectra of QN (Figure 5b) was almost three times higherthan in QL (Figure 5a), indicating more entrapment of the drug in QN. Additionally, a spectrum of Ca (calcium) was observed in QN which was absent in QL as liposomes were treated with calcium chloride for the formation of nancochleates. A spectrum of Cu (Copper) was seen in both images (Figure 4) as the samples were placed on a carbon-coated copper grid for analysis. The results were in accordance with the reports given by Alamet al. (2016) [56].

### 3.6. DSC of Quercetin and QN

**Figure 6:**
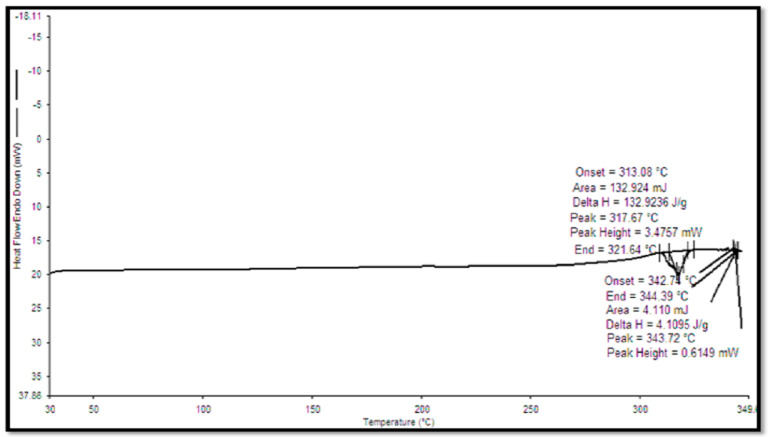
DSC of pure quercetin.

**Figure 7:**
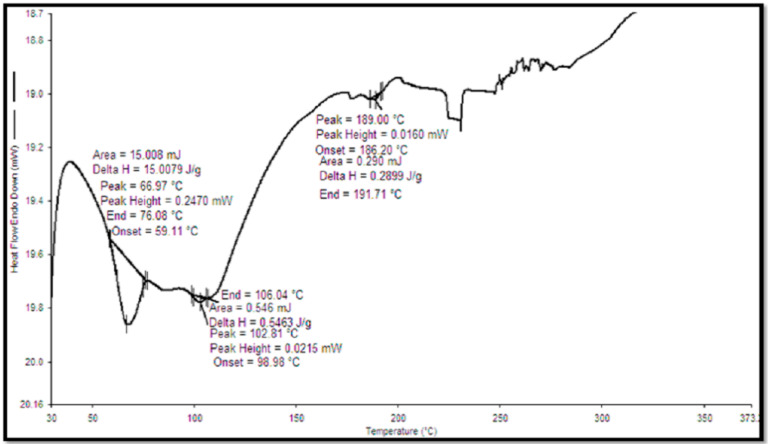
DSC of quercetin loaded nanocochleates.

DSC studies were performed for lyophilized quercetin-loaded nanocochleates to confirm the entrapment of quercetin in QN. DSC thermogram verified the physical nature of the drug in the formulation. A sharp endothermic peak of pure quercetin was observed at 317.67 °C (Figure 6) [57]. The elimination of the peak of a drug in the DSC thermogram of formulation (Figure 7) was the indication of the entrapment of the drug in the formulation [58].

### 3.7. In Vitro Release Study

**Figure 8:**
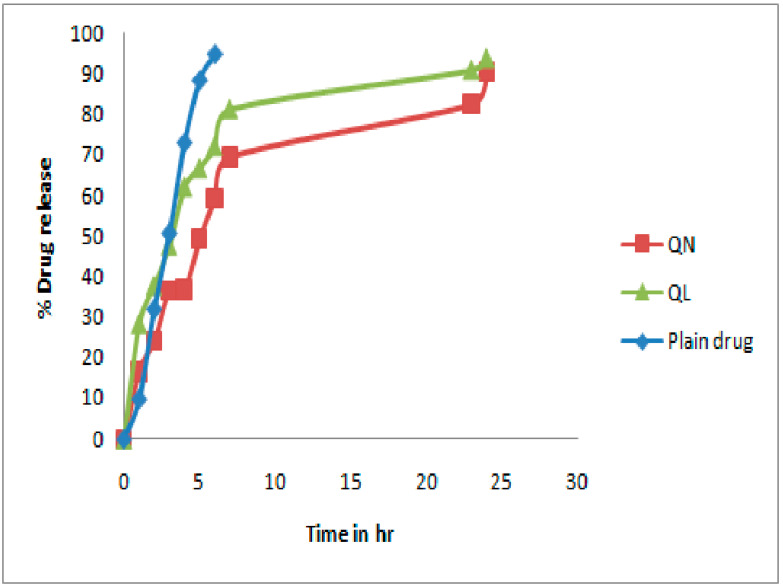
In vitro quercetin release.

In vitro release of quercetin was performed by the dialysis bag diffusion method in phosphate-buffered saline solution (pH 7.4). The release of quercetin from liposomes and nanocochleates in trial QL7 and QN3, respectively, was compared with the pure quercetin solution. As observed from Figure 8, the burst release of quercetin from QL7 and QN3 was noted within 1 h, which might be the result of the trapped drug being released on the surface of the formulations [59]. Almost 100% of the drug was released from the pure quercetin solution within 4 h, whereas QL and QN released 47% and 39% of the drug in 4 h, respectively. Compared to liposomes, nanocochleates showed more controlled release due to their coiled structure.

### 3.8. Degradation Studies of Quercetin in the Presence of Rat Liver Homogenate (S9G)

**Figure 9:**
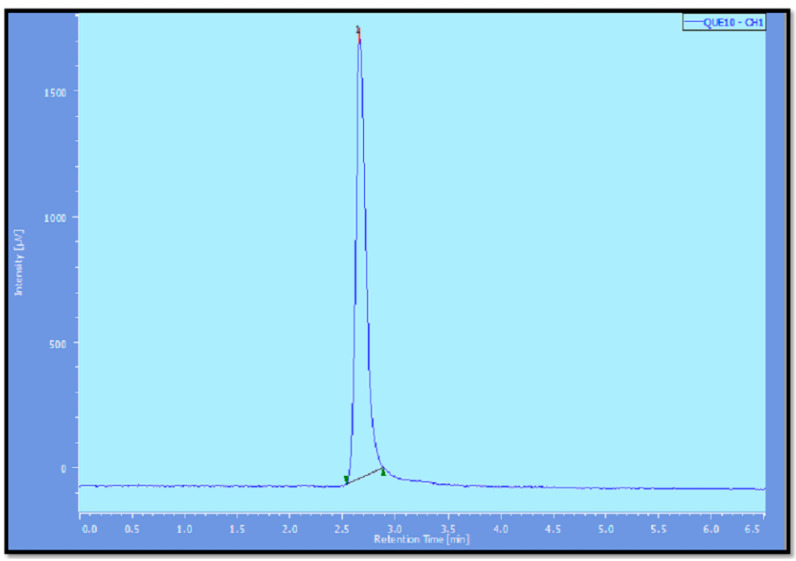
HPLC of pure quercetin.

**Figure 10:**
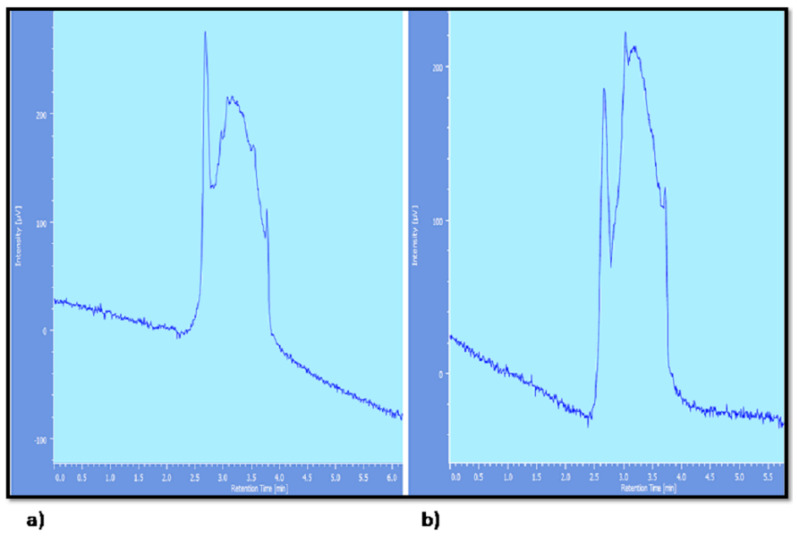

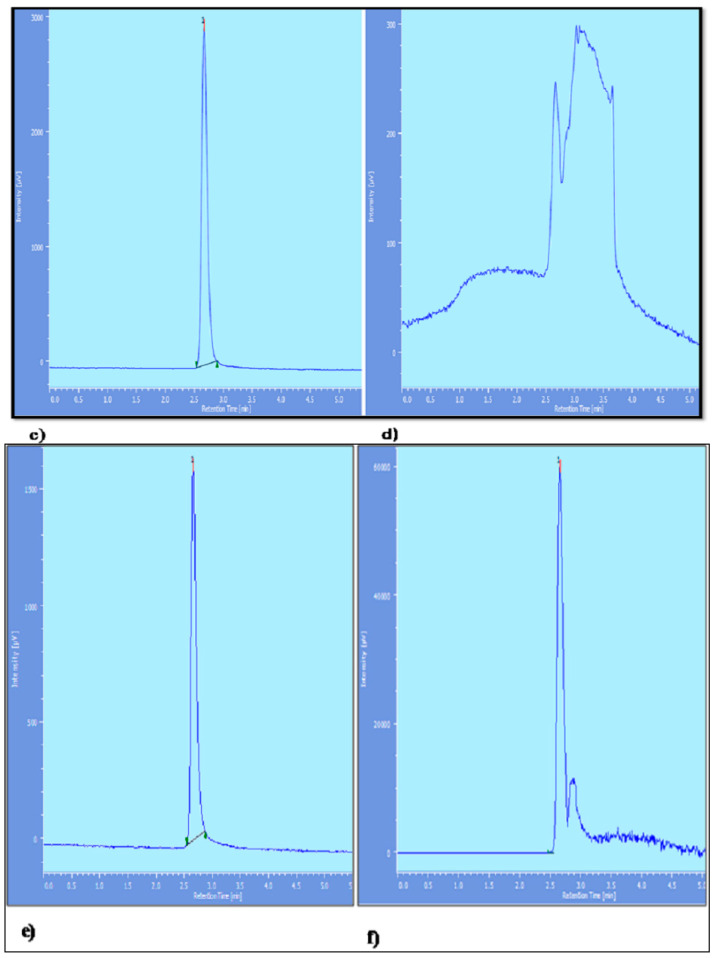
HPLC chromatogram of quercetin degradation after (**a**) 1 h and (**b**) 8 h.from plain solution; HPLC chromatogram of quercetin-loaded liposomes after (**c**) 1 h and (**d**) 8 h. HPLC chromatogram of quercetin-loaded nanocochleates after (**e**) 1 h and (**f**) 8 h.

Limited bioavailability of quercetin was due to its degradation in the presence of hepatic enzymes [60]. To mimic the in vivo conditions, rat liver homogenate (S9G) was added to the medium. To study the stability of quercetin released from pure quercetin solution, liposomes and nanocochleates, aliquots were withdrawn after a 1 h interval and 8 h interval and were injected into the HPLC System (JASCO LC–NET II/ADC, Japan). As seen from the HPLC chromatogram (Figure 9), theretention time of quercetin was found to be 2.6 in the absence of liver homogenate. In the presence of rat liver homogenate, HPLC chromatogram of pure quercetin solution showed an additional peak along with a peak of quercetin within 1 h (Figure 10a). The intensity of the additional peak increased while the intensity of the peak representing quercetin decreased at the end of the 8 h (Figure 9b). As seen from Figure 10c,e, the HPLC chromatogram of quercetin released from the liposomes and nanocochleatesafter1 h showed a characteristic peak of quercetin (RT = 2.6 min). The additional peak was observed at the end of the 8 h in both the liposomes and nanocochleates, but there was a significant difference regarding intensity (Figure 10d,f). This additional peak might be caused by the metabolism of quercetin in the presence of rat liver homogenate (enzymes that might be present in this homogenate lead to the conversion of quercetin into its metabolites). The intensity of the metabolite peak was negligible in the case of nanocochleates because the drug was protected due to the unique structure of the cochleates, which was uniform solid layer. Constituents trapped within the inner walls of the cochleate structure remain protected, irrespective of the exposure of the outer layers of the cochleates to enzymes or the harsh environment [61]. Thus, it could be concluded that quercetin could be well protected from the harsh environment inside the body, leading to a long residence time and can lead to its increased efficacy if it was entrapped in nanocochleates.

### 3.9. Cytotoxicity Study

**Figure 11:**
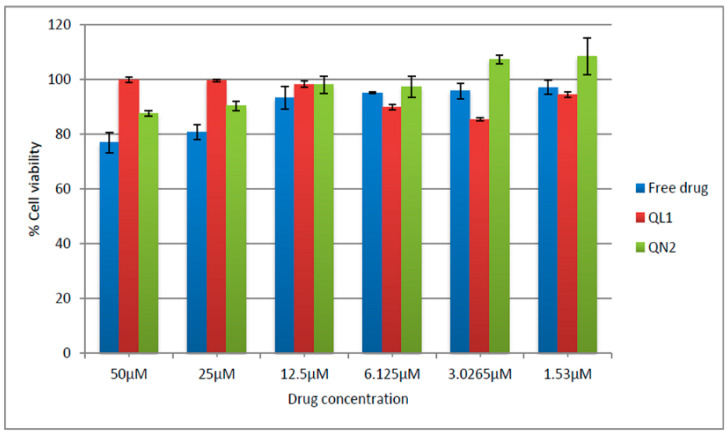
Comparative cytotoxicity study of free quercetin, quercetin-loaded liposomes and quercetin-loaded nanocochletaes. (*n* =3). Cell viability was found in the order of nanocochleates > liposomes > pure quercetin.

To study the biocompatibility of the developed formulations, cytotoxicity studies were carried out on fibroblast L929 cell lines. These cells had been used by the International Organization for Standardization (ISO) to carry out cytotoxicity studies [62]. The cut-off recommended by the ISO 10993-5: 2009 (Biological evaluation of medical devices part-5: Tests for in vitro cytotoxicity) for cytotoxicity was 70% [63]. For the current study, this limit was also considered acceptable. Few scientists conducted similar studies, and they considered 50% cell viability anacceptablelimit [64,65]. There was no remarkable reduction in the percentage of cell viability even whenincreasing the concentrations of the free drug, liposomes and nanocochleates as denoted in Figure 11. The viability of the cells was more than 78% in all the samples tested, which was well above the recommended cut-off limit. Hence, it could be concluded that liposomes and nanocochleates were biocompatible at varied concentration ranges.

### 3.10. In Vitro Anticancer Activity

**Figure 12:**
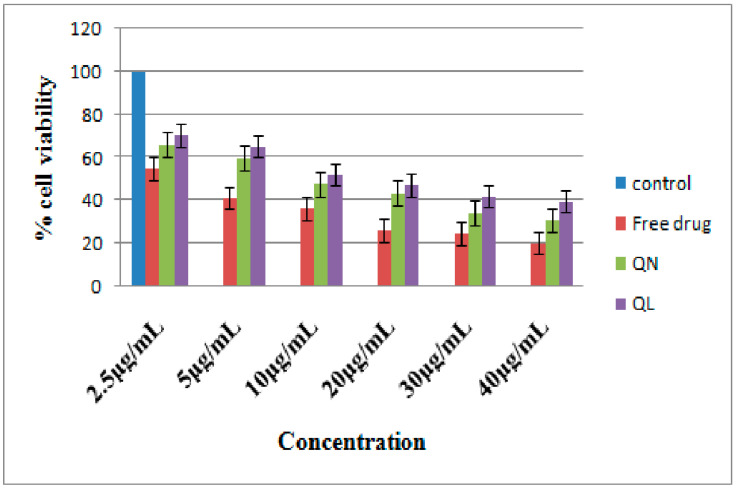
Comparative in vitro anticancer activity of pure quercetin, quercetin-loaded liposomes and quercetin-loaded nanocochleates on human mouth cancer cells 5000 KB studied using MTT assay (*n* =3).

Quercetin is a flavonoid and has various properties such asfree-radical scavenging activity, antioxidant potential and a direct, pro-apoptotic influence ontumor cells. Furthermore, itis capable of blocking the growth of numerous human cancer cell lines at diverse phases of the cell cycle [66]. In the present investigation, in vitro anticancer activity of pure quercetin and its formulations, QN and QL, wasproven using human mouth cancer cell lines 5000 KB. As illustrated in Figure 12, the growth inhibitory 50% (GI50) concentration of pure quercetin was found to be 5 µg/mL as it was in direct contact with cancerous cells. Though quercetin exhibited better anticancer potential, its efficacy was reduced due to poor oral bioavailability and degradation. Hence, there was a need for the development of a targeted drug delivery system that would protect the drug and release the drug for a prolonged period of time at the desired site. Thus, the rationale for the development of quercetin-loaded nanocochleates was proven. QN showed better anticancer activity than QL as seen from the GI50 concentrations of 10 and 20 µg/mL, respectively. This superior activity of QN might be attributed to perturbations and the reordering of cancerous cells which have negatively charged lipids upon contact with the calcium-rich highly ordered and linear (rod-shaped) structure of QN. On the other hand, an alternate mechanism might be phagocytosis, which causes encochleated drug to be absorbed by the cell after fusion with endocytic vesicles, subsequently controlling the release of the drug [67,68]. These results were in agreement with the research carried out by Nadaf and Killedar (2018). These researchers reported that calcium ions were responsible for enhanced membrane fusion and phagocytosis. Calcium ions act as a fusing agent and initiate the perturbations of the contact area, thus accelerating themembrane fusion process, resultingin increasedavailability of the drug to the targeted cells. Enhanced permeation and retention (EPR) effect of calcium rich nanocochleates was also reported [36]. Thus, nanocochleates could be successfully utilized as a novel carrier for the delivery of quercetin.

### 3.11. Stability Studies of Developed Formulations

**Figure 13:**
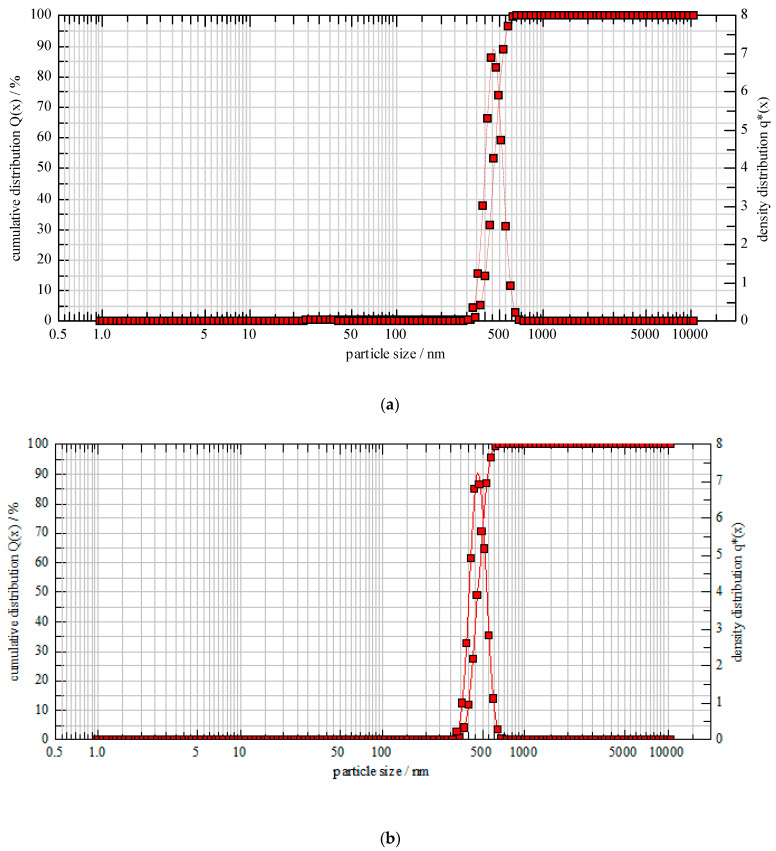
The particle size (cumulative distribution vs.particle size) of quercetin-loaded (**a**) liposomesamd (**b**) nanocochleates after three months of studying the stability.

**Figure 14:**
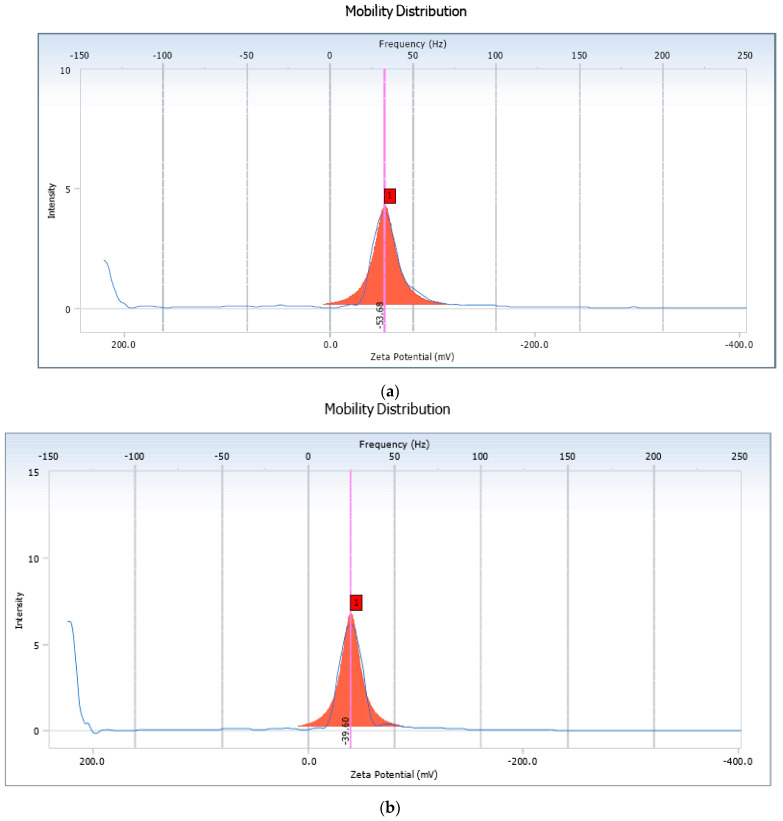
Zeta Potential of quercetin-loaded (**a**) liposomes (**b**)nanocochleates after three months of studying the stability.

The stability of lyophilized quercetin-loaded liposomal and nanocochleate formulations was tested at 5 ± 3 °C for 3 months. It was observed that lyophilized liposomal formulation was not stable, it formed agglomerates due to the fusion of lipids while lyophilized nanocochleates were stable, i.e., in the powder form. From Table 6, it can be seen that there was a significant reduction in the %EE of liposomes loaded with quercetin which might be due to leakage of the drug from the vesicles. An increased particle size of the liposomes after 3 months indicated the fusion of vesicles upon storage (Figure 12). Quercetin-loadednanocochleates did not show any significant change in these parameters after storage (Figure 13). The zeta potential of the liposomes and nanocochleateswasfound to be −53.68 and −39.60, respectively (Figure 14). Thus, it can be concluded that nanocochleates were more stable than liposomes.

## 4. Conclusions

Phospholipids are the main components of the cellular membrane and are well tolerated by the body. Phospholipids have the tendency to form liposomes which can be effective nanocarriers for the delivery of drugs, but they possess certain limitations such as the leakage of drugs from the vesicles, instability afterprolongedstorage and anaqueous core. These limitations can be overcome by the formulation of nanocochleates. The developed nanocochleates showed a significant difference in encapsulation efficiency and enabled thecontrolled release of quercetin for 24 h. Poor bioavailability of quercetin is due to its degradation by hepatic enzymes. To mimic these invivo conditions, rat liver homogenate (S9G) was added to the release medium, where quercetin was found intact and well protected due to the unique structure of the nanocochleates compared to the plain quercetin solution and liposomes. The formulations i.e quercetin loaded liposomes and nanocochleates were found to be nontoxic on L929 fibroblast cell lines. Thus, these were biocompatible. In vitro anticancer studies on human mouth cancer 5000 KB cell lines demonstrated thatnanocochleate formulation was superior to liposomes. Stability studies also indicated that nanocochleates retained their integrity and were found to be more stable, whereas liposomes fused and caused entrapped drug leakage. These findings can be further proved by invivo and clinical studies. Thus, it can be concluded that nanocochleates can act as a better alternative toliposomes for the delivery of drugs with poor stability and bioavailability, thus improving their therapeutic efficacy.

## Figures and Tables

**Table 1:** Formulation of quercetin-loaded liposomes.

Formulation	DMPG (mg)	Cholesterol (mg)	Quercetin (mg)
QL1	30	10	5
QL2	30	10	10
QL3	30	10	15
QL4	50	10	5
QL5	50	10	10
QL6	50	10	15
QL7	70	10	5
QL8	70	10	10
QL9	70	10	15

**Table 2:** Formulation of nanocochleates from optimized liposomal formulation.

Formulation	DMPG (mg)	Cholesterol (mg)	Quercetin (mg)
QN1	30	10	5
QN2	50	10	5
QN3	70	10	5

**Table 3:** ANOVA results for entrapment efficiency and particle size.

Experimental Response for Entrapment Efficiency (%)						
Source	Sum of Squares	Df	Mean Square	F Value	*p*-value	
Model	1293.09	8	161.64	6.366 × 10^7^	Prob > F	Significant
A-A	400.06	2	200.03	6.366 × 10^7^	<0.0001	
B-B	907.62	2	453.81	6.366 × 10^7^	<0.0001	
AB	180.38	4	45.09		<0.0001	
Pure Error	0.000	5	0.000		<0.0001	
Cor Total		13				
**Experimental Response for Particle Size (nm)**						
Source	Sum of Squares	Df	Mean Square	F Value	*p*-value	
Model	85,538.86	8	10,692.36	6.366 × 10^7^	<0.0001	Significant
A-A	50,304.12	2	25,152.06	6.366 × 10^7^	<0.0001	
B-B	17,901.63	2	8950.82	6.366 × 10^7^	<0.0001	
AB	24,594.72	4	6148.68	6.366 × 10^7^	<0.0001	
Pure Error	0.000	5	0.000			
Cor Total	85,538.86	13	10,692.36			

**Table 4:** Evaluation of liposomes loaded with quercetin.

Formulation	EE %	Vesicles Size (nm)	Zeta Potential (mV)	Appearance
QL1	70.5 ± 5.23	213 ± 3	−45.32	Spherical
QL2	55 ± 3.23	345 ± 2	−47.34	Spherical
QL3	35 ± 4.35	233 ± 4	−48.65	Spherical
QL4	72.4 ± 5.34	150 ± 2	−53.65	Spherical
QL5	66 ± 2.34	132 ± 5	−55.34	Spherical
QL6	59 ± 1.35	145 ± 3	−56.25	Spherical
QL7	74.2 ± 2.34	111.06 ± 2	−40.33	Spherical
QL8	65 ± 1.56	238 ± 3	−46.24	Spherical
QL9	58 ± 2.54	324 ± 6	−51.22	Spherical

EE and vesicle size parameters readings were taken in triplicate (Mean ± SD, *n* = 3).

**Table 5:** Evaluation of nanocochleates loaded with quercetin.

Formulation	% EE	Particle Size (nm)	Zeta Potential (mV)
QN1	78.2 ± 4.23	670 ± 3	−39.54
QN2	85 ± 3.25	544 ± 2	−26.32
QN3	88.62 ± 4.20	502 ± 4	−18.52

**Table 6:** Stability data of liposomes and nanocochleates.

Formulation	% EE		Particle Size (nm)		Zeta Potential (mV)		Appearance	
	0 Day	90 Day	0 Day	90 Day	0 Day	90 Day	0 Day	90 Day
QL7	74.2 ± 2.34	66 ± 2.21	111.06 ± 2	454.42 ± 2.8	−40.33	−53.68	Spherical	Spherical
QN3	88.62 ± 4.20	87.23 ± 3.24	502 ± 4	460.67 ± 3.33	−18	−39.60	Rod-shaped	Rod-shaped

## Data Availability

Not applicable.
